# Overall Proportion of Total DNA Consistent with an Individual Briefly Handling a Firearm

**DOI:** 10.3390/genes14122127

**Published:** 2023-11-25

**Authors:** Rachel Oefelein, Sarah Cresswell, Carney Matheson, Tania Jean-Louis

**Affiliations:** 1 DNA Labs International, Deerfield Beach, FL 33441, USA; rachel@dnalabsinternational.com (R.O.); tania@dnalabsinternational.com (T.J.-L.); 2 School of Environment and Science, Griffith University, Nathan, QLD 4111, Australia; c.matheson@griffith.edu.au

**Keywords:** DNA, firearms, persistence, activity level propositions, STRmix™, evaluative reporting

## Abstract

In forensic investigations, DNA profiles are routinely obtained from firearms evidence and alternative hypotheses may be proposed for consideration on the activity level. DNA profiles found to be consistent with the DNA profile of a specific individual could be a result of directly handling the firearm or other modes of transfer of DNA. Sixteen law-enforcement-owned firearms were evaluated with samples collected from the frame and slide area, the trigger and trigger guard area, and the front and rear sights after brief handling by laboratory personnel. Twenty-two out of forty-eight samples resulted in DNA profiles suitable for comparison, of which six resulted in likelihood ratios (LR) that demonstrated support for the hypothesis that included the brief handler as a contributor to the DNA profile obtained from the sample. Five of these samples were obtained from the frame and slide and one was from the trigger and trigger guard area. None of the DNA profiles obtained from the sights supported the inclusion of the brief handler as a contributor to the DNA profile. Gaining knowledge and supporting data on the nature of DNA profiles typically obtained from both owners and brief handlers can be useful for the purposes of evaluative reporting when considering results obtained from firearm evidence.

## 1. Introduction

Throughout the world, crime involving firearms is an ongoing issue with estimates of beyond 1 billion firearms throughout, approximately 85% of which are in civilian hands [1,2]. In the United States of America, there are more guns than people, with an average of approximately 120 firearms for every 100 people [1,2]. With a mere 4% of the global population, the United States manages to account for approximately 40% of all civilian firearms [1,2]. Further complicating matters is the range in types of firearms by country as a result of varying degrees of laws and limitations on the types of firearms that can be owned, i.e., automatic, versus semiautomatic, etc. With the prevalence of firearms throughout the world combined with the fact that firearms are inherently weapons, it is no surprise that firearms of all types are often submitted as evidence for DNA processing. As such, some research into the DNA collected from firearms and ammunition has already been conducted [3,4,5,6,7,8,9,10]. However, given the large quantities of firearms in the world, combined with their frequent association with crime, it is surprising that more research has not been published to date.

As the sensitivity of DNA testing has increased throughout the years, the question has changed from can a DNA profile be obtained to how did that DNA get there? This also becomes a question for the court as they are tasked with determining if the presence of a DNA profile has the potential to corroborate the implication of an individual in an alleged crime. On occasion, a hypothesis may be proposed that an individual handled a firearm; however, it was very brief. For example, an individual may have briefly handled a firearm to render the weapon safe. Law enforcement personnel, as a routine protocol, will render a firearm safe upon collection. Additionally, a civilian may also render a firearm safe or briefly handle the firearm for other reasons. The following question remains: what quantity of DNA may be expected to be deposited when an individual briefly handles a firearm, and how does this quantity of DNA compare to the quantity of DNA obtained from an owner of a firearm. It may be useful to ascertain if the profile obtained is consistent with the statements made by the persons of interest in relation to the case. Furthermore, when law enforcement has also interacted with the weapon, the presence of their DNA may also be informative in the evaluation of the evidence. When any type of evaluative statements are made in a report, the report falls into a category of evaluative reporting [11]. Evaluative reporting requires statistical calculations and research to support the statements made. There are guidelines that have been established on an international level [11,12] as well as guidelines that are in draft form in the United States [13]. However, universally, these standards agree that there must be research data relevant to the propositions in order to make an evaluation; hence, the lack of research data poses a problem in the implementation of evaluative reporting [14]. This research aimed to fill one of the gaps in the research data.

## 2. Materials and Methods

This research involved calculating the yield of DNA obtained from briefly held firearms and comparing the DNA profiles obtained to those of the owner of the firearm and an individual who briefly handled the firearm. All firearms used in this research were law enforcement owned pistols, whereas the owner was the sole owner of the firearm. Using law-enforcement-owned firearms and having only qualified scientists and law enforcement personnel participating in the study enabled the assurance of the safety of this research. A second individual, laboratory personnel, briefly handled the firearm for approximately 15 s. Seven individuals were utilized as the brief handlers. Handling the firearm included touching the exterior portions of the firearm by means of the gun being passed to the handler requiring contact with the frame and then holding the firearm by the grip. Contact was made with both hands on the firearm. The sampling of all firearms was over the course of several weeks based upon the availability of the firearm owners and handlers.

Known standards (oral swabs) were collected from the firearm owners as well as the brief handlers of the firearm. The firearm/owners of each firearm were assigned A-P designations for the purpose of this study. Buccal swabs were collected from each of the owners and handlers. Firearms were sampled with a single wet swab followed by a dry swab separately from the trigger and trigger guard area, the front and rear sights, and the frame and slide. Samples were processed by cutting the cotton swab material from the swab stick and depositing the cuttings into extraction tubes. Cotton swabs manufactured by Puritan were used for sample collection in conjunction with sterile disposable scalpels for removing the cotton swab material for extraction [10].

Samples were processed using a manual extraction method. Each sample was lysed with 400 μL of digest buffer (5N NaCL Solution), 30 μL of 10 mg/mL Proteinase K, and 20 μL of 1.0 M DTT (450 μL total) with overnight incubation at 56 °C. The samples were vortexed, centrifuged, and the substrates were removed via spin basket centrifugation. Subsequently, phenol/chloroform extraction was employed. The final elution volume was 30 μL. All samples were quantified using the Promega PowerQuant^®^ system. The final elution for the manual extraction process was approximately 28 μL after using 2 μL for quantitation. The total nanograms (ng) obtained was calculated by taking the value in ng/μL obtained from the Promega PowerQuant^®^ system and multiplying that amount by 30 [10]. The DNA yields were obtained and whether the values met the routine cut-off for polymerase chain reaction (PCR) amplification for casework was confirmed.

Regardless of the quantity of DNA obtained, all samples were amplified, and sample concentration was not utilized. Samples were amplified using Applied Biosystems GlobalFiler™ (Thermo Fisher Scientific, Waltham, MA, USA) or the Promega PowerPlex^®^ DNA profiling Fusion 6C (Promega™, Madison, WI, USA) systems. Both quantitation and amplification were automated on a TECAN Freedom EVO 150 system. Normalization was optimized for a 1.0 nanogram (ng) optimal input regardless of the system per the results of the laboratory’s existing protocols. Both DNA profiling systems have comparable sensitivity per the laboratory’s internal validation and meet the Combined DNA Index System’s (CODIS) core loci requirements [15,16]. Kit selection was made based on reagent availability and the two systems most commonly used in the laboratory. Amplification, using the manufacturer’s guidelines, was followed by capillary electrophoresis on an Applied Biosystems™ 3500xL Genetic Analyzer (Thermo Fisher Scientific, Waltham, MA, USA). The data were collected with 3500xL Series Data Collection software v.4 and analyzed using GeneMapper^®^ ID-X software v1.4 [10]. Whether the developed profile was suitable for comparison was evaluated. The interpretation of these data followed the operational guidelines, thresholds, and cut-offs of a standard accredited forensic DNA laboratory in the United States [10].

STRmix™ v2.6.02 software was used for mixture deconvolutions and likelihood ratio (LR) generation. The reported LR was derived from the Expanded Federal Bureau of Investigation (FBI) DNA population database (2016) for the Caucasian, Southeast Hispanic, Southwest Hispanic, and African American/Bahamian/Jamaican populations. The stratified LR, which uses the four noted population groups, was chosen for reporting. The value reported is an estimate that accounts for possible co-ancestry between individuals within racial sub-populations, sampling uncertainty of the allele frequencies, and variability within the STRmix^™^ deconvolution process.

First, the DNA profiles were considered if they originated from the owner of the firearm and N−1 unknown individuals (where N is the estimated number of contributors in the mixed DNA profile), or if the DNA profile originated from N individuals [10].

**Hypothesis** **1 (H1):** Owner and N Individuals−1.

**Hypothesis** **2 (H2):** N Individuals.

The second set of hypotheses considered whether the DNA profile originated from the handler of the firearm and N−1 unknown individuals (where N is the estimated number of contributors in the mixed DNA profile), or the DNA profile originated from N individuals.

**Hypothesis** **1 (H1):** Handler and N Individuals−1.

**Hypothesis** **2 (H2):** N Individuals.

The final set of propositions considered conditioned the owner of the firearm, considering whether the DNA profile originated from the handler of the firearm, the brief handler of the firearm, and N−2 unknown individuals (where N is the estimated number of contributors in the mixed DNA profile), or whether the DNA profile originated from the owner of the firearm and N−1 individuals. This analysis was only carried out for DNA profiles that indicated mixtures of at least two individuals as this proposition set would not be feasible for the single-source profiles.

**Hypothesis** **1 (H1):** Owner, Handler, and N Individuals−2.

**Hypothesis** **2 (H2):** Owner and N−1 Individuals.

The study was approved by the Human Research Ethics Committee of Griffith University (GU Ref No: 2022/275 approved 15 May 2022). Informed consent was obtained from all subjects involved in the study.

## 3. Results

### 3.1. Quantitation

Handling of these firearms happened subsequently to a previous firearm study [10], which may have resulted in a loss of the initial yield of DNA of the owner of the firearm as they did not have days of handling the firearm in the interim to establish a deposit of their DNA. However, given the smooth surface of the firearm, persistence of DNA may be limited inherently. Additionally, swabbing of a firearm will not remove all biological material that is present. This is evident by the quantities of DNA obtained in this study and the DNA typing results.

Quantitation of a sample plays a critical role in the DNA testing process in determining if DNA is present, to determine if there is enough DNA present to move forward with amplification, and to calculate the normalization of the sample. The total quantity of human DNA in nanograms (ng) was quantified. Y-DNA quantities did not play a critical role in this research given the non-sexual component of the firearm evidence, and as such have not been included in this summary [10]. Frame and slide area samples resulted in the highest yields as expected given the largest surface area sampled of all three sample types collected. Average total autosomal DNA yields by areas were 0.78 ng for the frame slide area, 0.03 ng for the sights, and 0.05 ng for the trigger and trigger guard. Utilizing a threshold of 0.001 ng/μL, 41% of the samples (*n* = 20) would have been stopped at quantitation from further processing per the laboratory’s casework quantitation cut-off for the PCR DNA profiling systems used.

**Figure 1:**
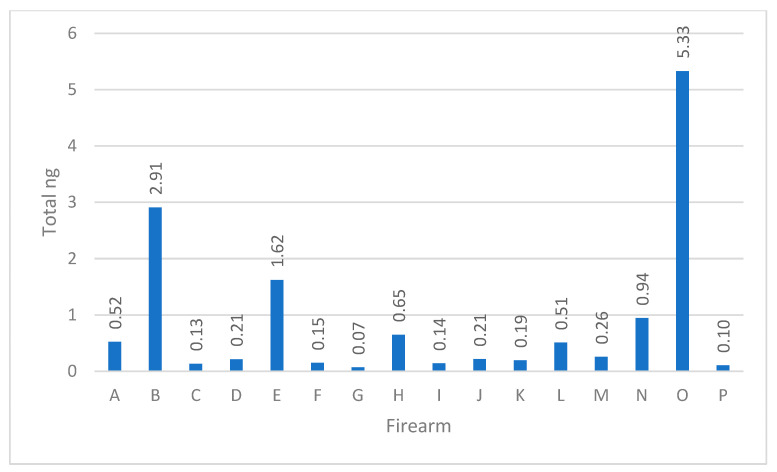
The sum of total DNA in nanograms recovered from each firearm.

The combined total DNA yield obtained from the three areas of each firearm was calculated [10]. The range in total human quantity of DNA spanned from around just 100 picograms (pg) to upwards of 5.33 ng, demonstrating how biology and human behavior can potentially play an important role in DNA yields (see Figure 1). For example, routine cleaning of the firearm, the method of cleaning of the firearm, and shedder status, to name a few, can impact the quantity of biological material that persists on a firearm and can subsequently be recovered. Firearms carried by law enforcement personnel are regularly stored in a holster, vault, or locked storage. Firearms in possession of criminals on the other hand may be kept in the waistband of clothing, inside pockets, or freely exposed in the individual’s environment. Increased skin to skin contact with a firearm will naturally result in higher quantities of DNA transfer. The routine cleaning of a firearm will decrease the persistence of cellular material [10].

### 3.2. Amplification and DNA Typing Results

The DNA typing results were assessed to determine if they would be considered inconclusive or suitable for comparison in casework, with approximately 45% of the 48 samples processed being suitable for comparison (*n* = 22). The suitability for comparison is based on the operational guidelines where, to even be eligible to be considered suitable for comparison, the minimum loci required is six non-sex-determining loci. Ten samples resulted in no observed DNA types above the analytical threshold. Unsurprisingly, nine out of the ten samples that did not yield a profile did not meet the internal cut-off to move forward for amplification, with the tenth sample just meeting the cut-off at 0.006 ng/μL. Fifteen of the remaining sixteen samples with observed DNA types deemed not suitable for comparison indicated the presence of at least one contributor. The sixteenth sample indicated a mixture of at least two individuals.

The vast majority of the samples deemed suitable for comparison included two-person mixtures with 54% of the DNA profiles suitable for comparison (*n* = 12). Single-source samples were second at approximately 36% (*n* = 8) and lastly, three-person mixtures at 9% (*n* = 2). In casework, DNA profiles obtained from firearms may also result in mixtures of four to five persons or more, demonstrating that the relatively low order of contributors observed in the DNA profiles for this sample set exhibits different behavior than what is typically observed in casework [10].

The presence or absence of a single allele, or even several alleles, consistent with a donor is not necessarily reflective of the presence of that donor, rather it could be coincidental allele sharing. Similarly, the absence of a single allele or even several alleles consistent with a donor is not necessarily reflective of the absence of that donor, rather it could be due to dropout. As such, the profiles deemed suitable for comparison were further evaluated utilizing probabilistic genotyping [10,17].

**Figure 2:**
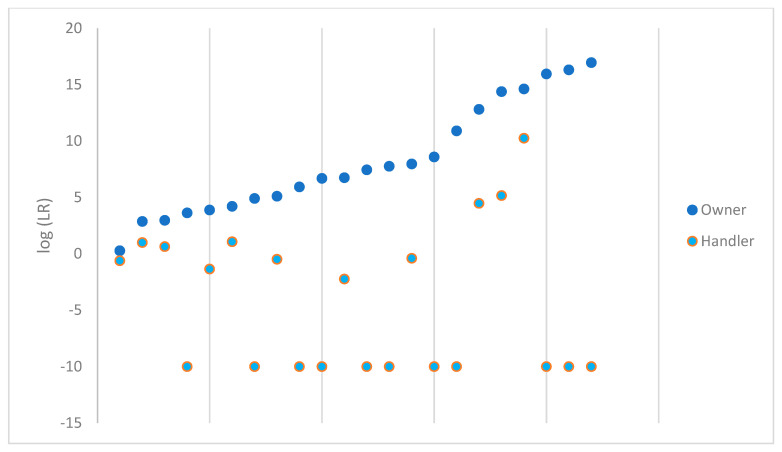
log(LR) obtained for the owner and handler of each firearm.

The probabilistic genotyping results from comparing each owner and handler are plotted in Figure 2 for the 22 samples that were considered suitable for comparison out of the 48 samples that were processed. For ease of viewing, these have been sorted from lowest value to highest value for the log(LR) of the owner. The owner and handler data points correspond vertically to the same sample. When a value of 0 was obtained, the log(LR) was plotted as −10.

There was some degree of support for the evidence under the proposition including the owner as a contributor to the DNA profile for each sample comparison. Not a single handler LR exceeded the value of the owner LR. Based on the Scientific Working Group on DNA Analysis Methods (SWGDAM) verbal scale recommendations, one handler LR would have fallen in the level of ‘very strong support’ [18]. Five additional samples supported the proposition that the handler is a contributor to the DNA profile obtained from the sample; the LR obtained for these five samples and their respective degree of support per the SWGDAM scale are shown in Table 1.

One of the limited support samples was sampled from the trigger and trigger guard area of the firearm, while the remaining five samples that supported H1 for the handler were collected from the frame and slide area. None of the samples from the sights supported H1 for the handler. This again brings up the discussion point of behavior of the firearm owner and the handler. The sights of the firearm may routinely come into contact with the body of the handler if the firearm is carried in the waistband of trousers and/or the undergarments of an individual. However, if a gun is holstered or briefly handled, frequent direct contact with the sights is not necessarily expected. Additionally, you would not expect to find much DNA on the trigger or trigger guard of a firearm unless the firearm had recently been fired. Any routine safe firearm handling instructions will instruct you to not put your finger on the trigger unless you intend to pull it.

**Figure 3:**
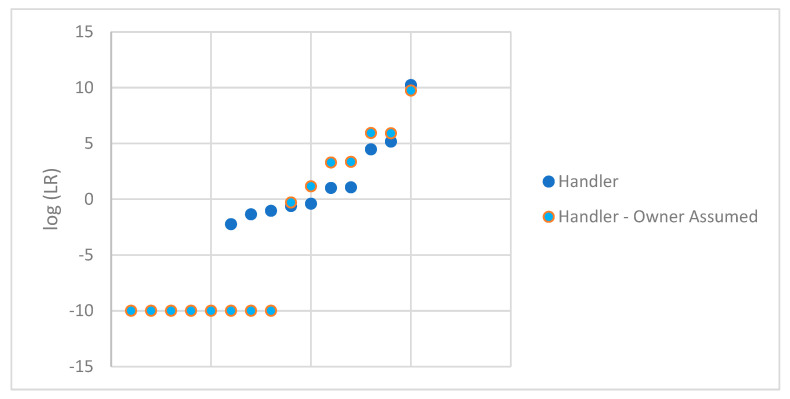
log(LR) obtained for the handler of each firearm with the owner conditioned.

The mixed DNA profiles that were suitable for comparison were further examined by conditioning on the owner of the firearm. In these circumstances, the ground truth is known, which allows for this conditioning. In casework, typically, the owner of the firearm will not be known, which would not allow for conditioning unless, at the very least, statistical support for the individual’s inclusion was first demonstrated. We assumed that the owner increased the number of samples that resulted in an LR of 0, with three additional samples resulting in 0 and the five samples that previously resulted in an LR of 0 remained 0. For the remaining samples, the LR increased by conditioning of the owner. This is to be expected since the genotype weights for the unknown contributor or contributors will typically increase with the more information that is provided. These samples were plotted in Figure 3 comparing the original log (LR) for DNA profile with the handler in H1 paired with the log (LR) for DNA profile with the handler in H1 with the assumed owner. For ease of viewing, the pair of LRs for each sample are aligned vertically. These LRs are also shown in Table 2.

**Figure 4:**
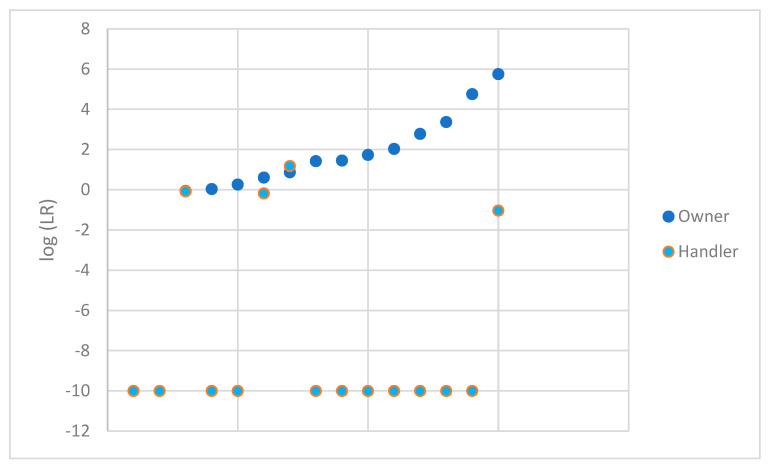
log (LR) obtained for the owner and handler of each firearm (profiles not suitable for comparison).

To avoid a non-response bias, profiles that were deemed not suitable for comparison were also evaluated. STRmix™ software has the ability to analyze profiles even when only a single allele type is present. Ten samples with no types observed above the analytical threshold were not considered further. Additionally, a sample with only a single type observed at Yindel was also eliminated from this additional analysis, as Yindel is not analyzed in STRmix™. This left fifteen additional samples to be analyzed using STRmix™. These data were plotted in Figure 4 in the same fashion as Figure 2. A summary of the data obtained is shown in Table 3 for these samples. As expected, given the limited nature of these profiles, the LRs for the DNA profile lean towards 1 considering the owner under H1. None of the LRs obtained when considering the owner exceeded 1 million. The vast majority of the LRs for the DNA profile considering the handler under H1 resulted in 0 or favored exclusion. A single comparison supported the inclusion of the handler with an LR of 15.

**Figure 5:**
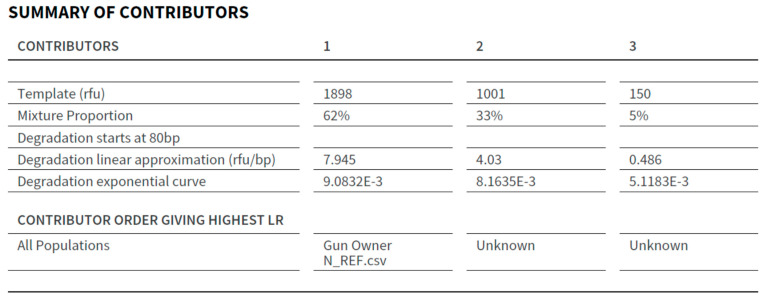
The Summary of Contributors section of the STRmix™ Report from the frame and slide of Gun N.

The STRmix™ output contains a summary of contributions with the approximate proportion of each of the contributors to the mixed DNA profile. The LR for the person of interest (POI) in H1 was assigned in the STRmix report to the contributor position that resulted in the highest LR for the POI. The contributor summary for the DNA profile obtained from the sample collected from the frame and slide of Gun N is shown in Figure 5. In this example, the highest LR supporting the proposition that the gun owner from N is a contributor to the DNA profile was in relation to contributor 1, approximately 62%.

**Figure 6:**
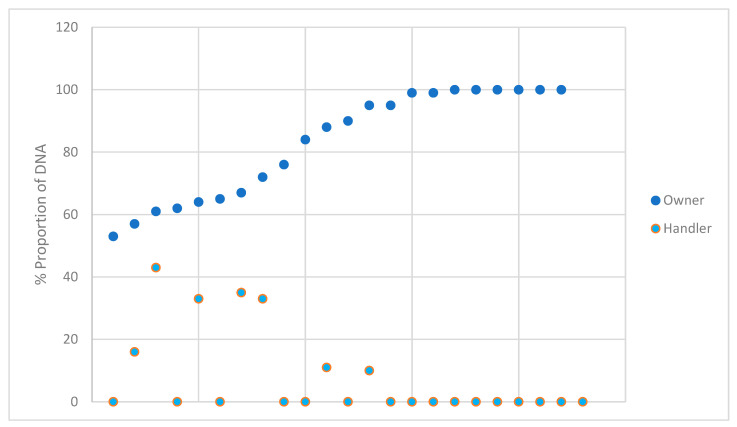
The estimated percent proportion of the DNA from the owner and brief handler of the profiles that were suitable for comparison.

The estimated proportions for each owner and handler for the DNA profiles that were deemed suitable for comparison were plotted and shown in Figure 6. The owner and handler data points correspond vertically to the same sample. The data from the samples not considered suitable for comparison were not included due to the vast majority of the types observed being only 1–3 allele types and the relatively low likelihood ratios obtained. None of the handler samples exceeded an estimate of 43% and none of the owner samples were estimated lower than 53%. During expert witness testimony, the question ‘is this individual the major or the minor DNA profile’ may be posed. When utilizing probabilistic genotyping, the DNA analyst may not necessarily assign the designation of major or minor; however, it is important to determine the approximate amount of DNA an individual is contributing to a DNA profile. The proportion of DNA is not necessarily reflective of high LRs, as a fully represented minor contributor will still produce a high LR when evaluated in the H1 proposition. Evaluating the proportion of DNA associated with the contributor position of the assigned LR may benefit the evaluation of alternate hypotheses.

## 4. Discussion

When processing firearm samples for DNA analysis, there may be multiple contributors to the DNA profile obtained. In this study, the highest yields of DNA and the highest percentage of DNA profiles deemed suitable for comparison were collected from the frame and slide area opposed to the front and rear sight area or the trigger and trigger guard area. When the owner of the firearm’s DNA profile is available for comparison, it can be used to condition the profile, providing additional information that can benefit the deconvolution of the remainder of the DNA profile. When the profile was deemed suitable for comparison, all analyses supported the inclusion of the owner of the firearm. Approximately 27% of the time, there was support for the DNA profile under the proposition that a brief handler of the firearm was a contributor to the DNA profile obtained; however, the results typically fell into the category of limited support [18]. Additionally, the brief handler LR never exceeded that of the owner LR, nor did the estimated proportion of the total DNA associated with the LR of the brief handler exceed that of the owner. If an individual, albeit as a result of alleged criminal activity or law enforcement intervention, briefly handles a firearm, these data may be used for the purpose of further analyzing that data for the purpose of evaluative reporting.

## Figures and Tables

**Table 1:** Summary of samples that supported the proposition that the handler is a contributor to the DNA profile.

Number of Contributors	Handler LR	Area	Level of Support
2	4.29E+00	Frame/Slide	Limited
2	1.00E+01	Trigger/Trigger Guard	Limited
2	1.16E+01	Frame/Slide	Limited
2	2.94E+04	Frame/Slide	Moderate
3	1.45E+05	Frame/Slide	Strong
3	1.73E+10	Frame/Slide	Very Strong

**Table 2:** log(LR) obtained for the owner and handler of each firearm by the sampled area.

Area	Total Auto (ng)	NOC	Firearm	Owner LR	Handler LR	Handler LR (Owner Assumed)	Owner Proportion	Handler Proportion
Sights	0.20	2	A	7.50E+03	4.42E−02	0.00E+00	64	0
Frame/Slide	2.61	2	B	2.01E+16	0.00E+00	0.00E+00	95	0
Trigger/Trigger Guard	0.10	1	C	7.74E+10	0.00E+00	-	100	0
Frame/Slide	0.16	2	D	3.81E+08	0.00E+00	0.00E+00	72	0
Frame/Slide	1.49	2	E	8.67E+15	0.00E+00	0.00E+00	95	0
Sights	0.02	1	E	4.24E+03	0.00E+00	-	100	0
Frame/Slide	0.12	2	F	5.57E+06	5.76E-03	0.00E+00	88	0
Frame/Slide	0.05	1	G	1.24E+05	0.00E+00	-	53	16
Frame/Slide	0.49	3	H	2.37E+14	1.45E+05	8.27E+05	84	11
Sights	0.04	1	H	4.80E+06	0.00E+00	-	100	0
Trigger/Trigger Guard	0.08	2	H	5.66E+07	0.00E+00	0.00E+00	61	0
Frame/Slide	0.11	1	I	9.21E+02	4.29E+00	-	65	35
Frame/Slide	0.16	2	K	1.59E+04	1.16E+01	2.25E+03	67	33
Sights	0.01	2	K	1.85E+00	2.40E-01	5.11E−01	99	0
Frame/Slide	0.37	2	L	6.33E+12	2.94E+04	8.58E+05	90	10
Frame/Slide	0.17	2	M	9.21E+07	3.96E−01	1.42E+01	76	0
Frame/Slide	0.73	3	N	4.12E+14	1.73E+10	5.52E+09	62	33
Trigger/Trigger Guard	0.14	2	N	7.43E+02	1.00E+01	1.94E+03	57	43
Frame/Slide	4.83	2	O	8.70E+16	0.00E+00	0.00E+00	99	0
Trigger/Trigger Guard	0.09	1	O	8.37E+05	0.00E+00	-	100	0
Frame/Slide	0.04	1	P	7.95E+04	0.00E+00	-	100	0
Trigger/Trigger Guard	0.06	1	P	2.77E+07	0.00E+00	-	100	0

**Table 3:** log (LR) obtained for the owner and handler of each firearm by the sampled area (profiles not suitable for comparison).

Area	Total Auto (ng)	NOC	Firearm	Owner LR	Handler LR
Trigger/Trigger Guard	0.09	1	A	0	0
Frame/Slide	0.22	2	A	5.59E+05	9.19E−02
Trigger/Trigger Guard	0.10	1	B	7.31E+00	1.51E+01
Trigger/Trigger Guard	0.02	1	D	8.73E−01	8.31E−01
Sights	0.01	1	F	1.08E+00	0
Sights	0.00	1	G	0	0
Trigger/Trigger Guard	0.01	1	G	1.07E+02	0
Sights	0.01	1	J	4.03E+00	6.50E−01
Trigger/Trigger Guard	0.01	1	K	1.81E+00	0
Sights	0.05	1	L	2.64E+01	0
Trigger/Trigger Guard	0.06	1	L	5.61E+04	0
Sights	0.03	1	M	5.39E+01	0
Trigger/Trigger Guard	0.04	1	M	5.97E+02	0
Sights	0.02	1	N	2.83E+01	0
Sights	0.05	1	O	2.31E+03	0

## Data Availability

The data presented in this study are available upon request from the corresponding author. The data are not publicly available due to privacy and ethical restrictions.
